# Nucleolar proteins Bfr2 and Enp2 interact with DEAD-box RNA helicase Dbp4 in two different complexes

**DOI:** 10.1093/nar/gkt1293

**Published:** 2013-12-18

**Authors:** Sahar Soltanieh, Martin Lapensée, François Dragon

**Affiliations:** Département des sciences biologiques and Centre de recherche BioMed, Université du Québec à Montréal, Montréal, Québec, Canada

## Abstract

Different pre-ribosomal complexes are formed during ribosome biogenesis, and the composition of these complexes is highly dynamic. Dbp4, a conserved DEAD-box RNA helicase implicated in ribosome biogenesis, interacts with nucleolar proteins Bfr2 and Enp2. We show that, like Dbp4, Bfr2 and Enp2 are required for the early processing steps leading to the production of 18S ribosomal RNA. We also found that Bfr2 and Enp2 associate with the U3 small nucleolar RNA (snoRNA), the U3-specific protein Mpp10 and various pre-18S ribosomal RNA species. Thus, we propose that Bfr2, Dbp4 and Enp2 are components of the small subunit (SSU) processome, a large complex of ∼80S. Sucrose gradient sedimentation analyses indicated that Dbp4, Bfr2 and Enp2 sediment in a peak of ∼50S and in a peak of ∼80S. Bfr2, Dbp4 and Enp2 associate together in the 50S complex, which does not include the U3 snoRNA; however, they associate with U3 snoRNA in the 80S complex (SSU processome). Immunoprecipitation experiments revealed that U14 snoRNA associates with Dbp4 in the 50S complex, but not with Bfr2 or Enp2. The assembly factor Tsr1 is not part of the ‘50S’ complex, indicating this complex is not a pre-40S ribosome. A combination of experiments leads us to propose that Bfr2, Enp2 and Dbp4 are recruited at late steps during assembly of the SSU processome.

## INTRODUCTION

The making of eukaryotic ribosomes is an intricate process that is highly conserved. Our knowledge of ribosome biogenesis comes mainly from studies in the budding yeast *Saccharomyces cerevisiae* (1–4). Ribosome biogenesis initiates within the nucleolus, continues in the nucleoplasm and terminates in the cytoplasm. This process involves ribosomal RNA (rRNA) transcription, processing, modification and assembly of rRNAs with ribosomal proteins, which leads to the synthesis of the small and large ribosomal subunits (40S and 60S) (1,2,5,6).

**Figure 1.**
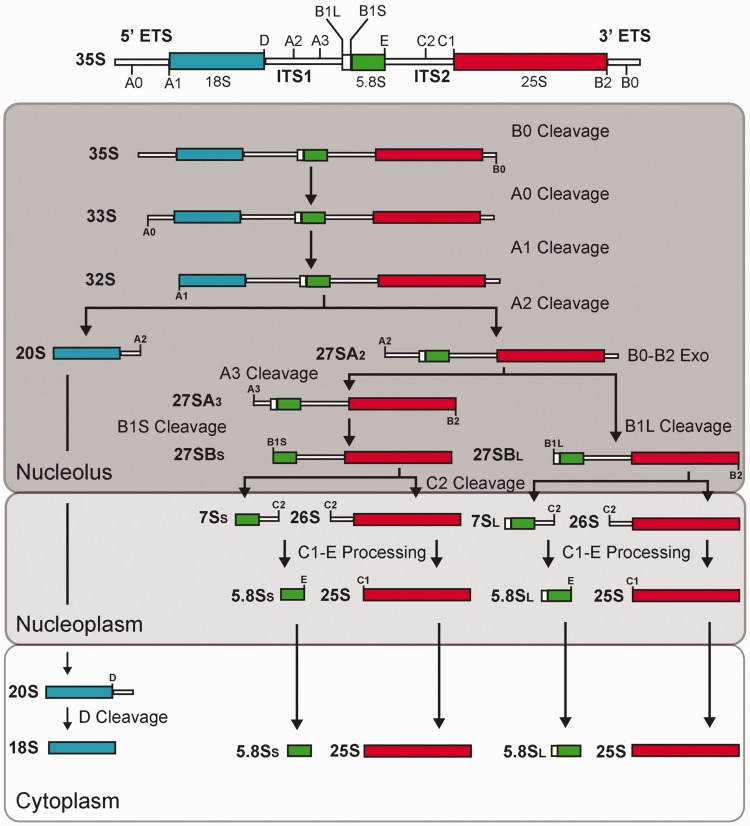
The pre-rRNA processing pathway in yeast. The structure of the 35S pre-rRNA (primary transcript) is shown on top. The rectangles represent cellular compartments in which different steps of the processing pathway take place. The pre-rRNA cleavage sites are indicated on the transcripts.

A key process in ribosome biogenesis is the production of mature rRNAs, the functional components of ribosomes (7). Yeast RNA polymerase I synthesizes a long precursor of 35S that encodes the 18S, 5.8S and 25S rRNAs, whereas the 5S rRNA is independently transcribed by RNA polymerase III (2,8). The 35S pre-rRNA is subjected to an orderly maturation process that requires about 200 *trans*-acting factors (1,6,8,9). In addition, tens of small nucleolar RNAs (snoRNAs) base pair transiently with pre-rRNAs and direct site-specific post-transcriptional modification of rRNAs. Very few snoRNAs are required for the endonucleolytic cleavages that remove spacer sequences from pre-rRNAs. In yeast, only U3, U14 and snR30 snoRNAs are essential for the cleavage reactions that lead to the production of 18S rRNA (2,10,11). The functionally active U3 ribonucleoprotein (RNP) is a very large complex of ∼80S called the small subunit (SSU) processome, which is formed at the 5′ end of nascent pre-rRNA and can be seen under the electron microscope (12,13). In yeast, the SSU processome is implicated in early pre-rRNA cleavages at processing sites A0, A1 and A2 (12,13; Figure 1). The SSU processome is an early pre-ribosomal particle that is necessary for maturation of the 18S rRNA: it contains the U3 snoRNA and about 72 proteins including ribosome biogenesis factors and ribosomal proteins (14). These proteins assemble and interact together to form the SSU processome. A number of studies identified the presence of sub-complexes of the SSU processome. These sub-complexes are called UtpA/tUTP, UtpB, UtpC, Mpp10, Rcl1/Bms1 and U3 snoRNP (15–25). However, proteins identified from these sub-complexes account for 43% of the proteins of the SSU processome, indicating that many proteins of the SSU processome have not yet been identified as components of a sub-complex (14,26). There are also studies showing that some of the sub-complexes of the SSU processome associate with the rRNA precursors in a hierarchical and stepwise manner (22,27,28).

Many of the non-ribosomal factors involved in rRNA maturation are RNA helicases. These enzymes are viewed as molecular motors that rearrange RNA structures in an energy-dependent fashion (29–35). However, some can rearrange RNA–protein complexes, and many could in fact be RNPases (30,36). DEAD-box protein Dbp4 is a RNA helicase that is phylogenetically conserved and essential for yeast viability; Dbp4 was first identified as a multi-copy suppressor of lethal mutations in the Y domain of U14 snoRNA (37). More recently it was shown that Dbp4 is required for the production of 18S rRNA and more specifically for the early cleavages at sites A0, A1 and A2 of the pre-rRNA (38). The C-terminal extension that flanks the catalytic core of Dbp4 contains a predicted coiled-coil motif, which is conserved in all Dbp4 orthologs (our unpublished observation). Because this motif is implicated in protein–protein interactions, Dbp4 might function in a complex with other protein(s). We found that Dbp4 associates with the essential nucleolar proteins Bfr2 and Enp2 (39–41). We also show that Bfr2 and Enp2 are implicated in the early cleavages leading to 18S rRNA production, and that Bfr2 and Enp2 associate with the U3 snoRNA and the U3-specific protein Mpp10. Sucrose gradient analyses and immunoprecipitation assays revealed that Dbp4, Bfr2 and Enp2 associate together in complexes of 50S and 80S. These proteins do not associate with the U3 snoRNA in the 50S peak; however, they interact with the U3 snoRNA in the 80S peak.

## MATERIALS AND METHODS

### Yeast strains and media

All conditional yeast strains and strains expressing tagged proteins were derived from YPH499 (*MAT*a*, ura3-52, lys2-80, ade2-101, trp1-Δ63, his3-Δ200* and *leu2-Δ1*) (42). We generated strain GAL::HA-BFR2 (alias YSS5) that expresses 3×HA-tagged Bfr2 under the control of the *GAL1* promoter, which was substituted for the natural promoter by chromosomal integration at the *BFR2* locus (43). Strain YSS5 was further engineered to produce 9×myc-tagged Enp2 expressed from its natural promoter (44): this new strain (YSS7) is hereafter referred to as the double-tagged strain. Strain GAL::ENP2-myc (alias YSS9) expresses C-terminally 9×myc-tagged Enp2 under the control of the *GAL1* promoter (43). Strain GAL::DBP4-HA expresses 3×HA-tagged Dbp4 under the control of the *GAL1* promoter (43). Strain AH109 was obtained from Clontech (*MAT*a*, trp1-901, leu2-3, 112, ura3-52, his3-200, gal4Δ, gal80Δ, LYS2::GAL1_UAS_−GAL1_TATA_−HIS3, GAL2_UAS_−GAL2_TATA_−ADE2 URA3:: MEL1_UAS_−MEL1_TATA_−LacZ MEL1*). The strains were grown in rich medium YPD (1% yeast extract, 2% peptone, 2% dextrose), YPGal (1% yeast extract, 2% peptone, 2% galactose) or synthetic minimal media (0.67% yeast nitrogen base) complemented with the proper dropout mix and appropriate carbon source.

### Two-hybrid analyses

The ORF encoding Dbp4 was amplified by polymerase chain reaction from genomic DNA isolated from yeast strain YPH499 following the procedure of Asubel *et al.* (45). Primers DBP4–forNco 5′-CAT GCC ATG GCC AAA AAA AAT AGA TTG AAC-3′ and DBP4–revXma 5′-CCC CCC GGG TTA ACC CTG GAT TAA TTT AGC TGT C-3′ were used, and the DNA fragment was cloned between the NcoI and XmaI sites of pGBKT7 (Clontech) to produce pGBK-DBP4. This plasmid was transformed into yeast strain AH109 and used as bait in a two-hybrid screen carried out with yeast genomic libraries (46). Plasmids pGAD–DBP4, pGAD–BFR2 and pGAD–ENP2 were prepared as described earlier, except that primer pairs DBP4–forXma 5′-CCC CCC GGG TAT GGC CAA AAA AAA TAG ATT GAA-3′ and DBP4–revXho 5′-CCG CTC GAG TTA ACC ATG GAT TAA TTT AGC TGT C-3′, BFR2–forXma 5′-CCC CCC GGG TAT GGA AAA ATC ACT AGC GGA TCA AAT TTC C-3′ and BFR2–revXho 5′-CGC CTC GAG TCA ACC AAA GAT TTG GAT ATC ATC GTT TTT AAC-3′, and ENP2–forXma 5′-CCC CCC GGG TAT GGT TTT GAA ATC TAC TTC CGC AAA TG-3′ and ENP2–revXho 5′-CGC CTC GAG CTA CAT ACC ACG GAA CGC ATT TTT G-3′ were used to amplify the ORFs of *DBP4*, *BFR2* and *ENP2*, respectively, and the DNA fragments were individually cloned between de XmaI and XhoI sites of pGADT7 (Clontech). The integrity of two-hybrid constructs was verified by automated sequencing at the McGill University and Génome Québec Innovation Centre.

The interaction between Dbp4 and various proteins was assessed by the yeast two-hybrid assay in strain AH109. To this end, pGBK–DBP4 was used as bait, and prey plasmids included pGAD–DBP4, pGAD–BFR2, pGAD–ENP2 and pGAD-NOP6. Bait and prey plasmids were simultaneously transformed into yeast strain AH109, and double transformants were selected onto SD–Trp–Leu agar plates (45). For each combination of bait and prey plasmids, transformants were first streaked onto a SD–Trp–Leu plate and after 3 days of incubation at 30°C, the cells were restreaked onto a SD–Trp–Leu–His plate. To increase the stringency of the two-hybrid assay, 3-amino-1,2,4-triazole (3-AT) was added to SD–Trp–Leu–His plates at concentrations of 2 mM or 20 mM. Empty bait or prey plasmids were used as controls.

### Antibodies

The antibodies used in this study are as follows: anti-HA mouse monoclonal antibody (mAb) (12CA5 hybridoma supernatant), anti-myc mouse mAb (9E10 hybridoma supernatant), anti-Mpp10 rabbit polyclonal (47), anti-Dbp4 rabbit polyclonal antibodies, anti-Tsr1 rabbit polyclonal (48), anti-MBP rabbit polyclonal antibodies (NEB), anti-Penta·His mouse mAb (QIAGEN) and anti-GST goat polyclonal antibodies (GE Healthcare).

The anti-Dbp4 antibodies were raised against recombinant Dbp4Δcat, which lacks most of the catalytic domain of Dbp4 to avoid cross-reaction with other DEAD-box RNA helicases. His-tagged Dbp4Δcat was produced in *Escherichia coli* BL21(DE3) pLysA from the pET23a(+) vector, a kind gift of T.H. King and M.J. Fournier (University of Massachusetts, Amherst, USA); this construct encodes a mutant derivative of Dbp4 lacking most of the catalytic domain due to elimination of the in-frame EcoRI fragment. His-tagged Dbp4Δcat was first isolated on a HisTrap column using the ÄKTApurifier, as recommended by the manufacturer (GE Healthcare). During elution, fractions of 500 µl were collected and peak fractions were pooled; recombinant Dbp4Δcat was further purified by electrophoresis in preparative sodium dodecyl sulfate (SDS) gels. These gels were subjected to reverse staining (49), and the 46-kDa band corresponding to Dbp4Δcat was excised, electro-eluted and concentrated (Microcon filters, Millipore). The purified protein was quantified with the Bio-Rad Protein Assay and stored at −80°C. Immunization of rabbits was carried out in-house at the Animal Care Facility.

### Immunoprecipitations

Immunoprecipitation experiments (IPs) were conducted with whole cell extracts (WCEs) prepared from exponentially growing cells. Cells were harvested by centrifugation, washed with sterile water and broken with glass beads in TMN100 buffer (25 mM Tris-HCl, pH 7.5, 100 mM NaCl, 10 mM MgCl_2_ and 0.1% NP-40). For RNase treatment, the WCEs were pre-incubated with 30 µg RNase A (Sigma) for 10 min at 37°C, and the mock experiments were incubated similarly, except that no RNase A was added. Thirty A_600_ units of cells were collected and after preparation of cellular extract, the equivalent of five A_600_ units were used for each IP experiment; when IPs were done to verify association of large RNA precursors, 30 A_600_ units were used. IPs were also carried out on fractions from sucrose density gradients: fractions 3, 4 and 5 were pooled together and formed the ‘50S’ peak, whereas pooled fractions 7 and 8 formed the ‘80S’ peak. Cell lysates were incubated with protein-A agarose beads (Roche) saturated with anti-Dbp4, anti-Mpp10, anti-HA or anti-myc antibodies. IPs were done at 4°C for 1 h on a Nutator, and immunoprecipitates were washed five times with 1 ml of TMN100 buffer. For protein analyses, the immunoprecipitates were either mixed with 2× SDS loading buffer or eluted with elution buffer (25 mM Tris-HCl, pH 7.5, 10 mM EDTA and 0.5% SDS) for 10 min at 65°C, and 2× SDS loading buffer was added afterwards. For RNA analysis, the immunoprecipitates were eluted with the elution buffer, extracted with phenol/chloroform and precipitated with ethanol. The precipitated RNA was either resuspended in 95% formamide or in 51% formamide and 17% formaldehyde to analyze the U3 snoRNA or large RNAs, respectively.

### Western blotting

Protein samples were separated by SDS-polyacrylamide gel electrophoresis, transferred onto a polyvinylidene difluoride membrane and subjected to immunodetection with anti-HA (1/100), anti-myc (1/100), anti-Mpp10 (1/10 000) or anti-Dbp4 (1/3000), anti-MBP (1/10 000), anti-His (1/1000) and anti-GST (1/1500), and the appropriate horse radish peroxidase-conjugated secondary antibodies were used (GE Healthcare). Immunoblots were revealed by chemiluminescence with the Immmobilon Western kit (Millipore).

### Northern blotting

To analyze precursor and mature rRNAs, total RNA was extracted with hot acidic phenol (45). To detect the U3 snoRNA in either sucrose gradient fractions or IP assays, RNA was isolated using phenol/chloroform extraction as described by Ausubel *et al.* (45). Large RNAs were separated on 1.2% formaldehyde-agarose gels and small RNAs were separated on 8% denaturing polyacrylamide gels. Northern hybridization was carried out with radiolabeled oligonucleotide probes complementary to the U3 snoRNA or to different rRNA precursors. The oligonucleotides used are as follows: anti-U3, 5′-CCA AGT TGG ATT CAG TGG CTC-3; 5′-A0, 5′-CGC TGC TCA CCA ATG G-3′; D-A2, 5′-GCT CTC ATG CTC TTG CC-3′; A2-A3, 5′-TTG TTA CCT CTG GGC CC-3′; anti-18S, 5′-CAT GGC TTA ATC TTT GAG AC-3′; anti-25S, 5′-CTC CGC TTA TTG ATA TGC-3′; anti-5.8S, 5′-GCG TTG TTC ATC GAT GC-3′; anti-U14 5′-CGA TGG GTT CGT AAG CGT ACT CCT ACC GTG G-3′. The mature 18S and 25S rRNAs were visualized by staining with GelRed™ (Biotium). Membranes were exposed to a phosphor screen and revealed with a Molecular Imager FX (Bio-Rad).

### Sucrose density gradients

WCEs were fractionated on 7–47% linear sucrose gradients as described by Lemay *et al.* (50), except that the lysis buffer was TMK100 (25 mM Tris-HCl, pH 7.5, 100 mM KCl, 10 mM MgCl_2_ and 0.1% NP-40). Sixteen fractions were collected with an ISCO density gradient fractionation system coupled to a UA-6 detector to produce continuous absorbance profiles at 254 nm. Eighty microliters of each fraction was used for protein analyses, and 200 µl used for RNA analyses.

### Pull-down assays

The ORFs encoding Bfr2, Dbp4 and Enp2 were cloned into the following plasmids: pMAL-c5 (NEB), pET-23a(+) (Novagen) and pGEX-4T-1 (GE Healthcare). Proteins were expressed in Rosetta™(DE3) pLysS cells (Novagen). Chloramphenicol and ampicillin were supplemented to the LB medium. Overnight cultures were grown at 37°C, then diluted and grown again to an A_600_ of ∼0.6 before induction of 1 mM IPTG. After 2–4 h of induction at 30°C, the cells were harvested, and the pellet was resuspended in lysis buffer (BugBuster®, Novagen). The MBP–Bfr2 extract was precipitated with ammonium sulfate (40%), and the pellet was resuspended in TMN100. The binding and elution of MBP or MBP–Bfr2 fusion protein was carried out according to pMAL protein fusion and purification system manual (NEB), using amylose magnetic beads (NEB). MBP–Bfr2-coated beads were incubated with Dbp4–His or GST–Enp2, washed with TMN100 and eluted with maltose. Pull-down experiments were also done in the presence of yeast total RNA isolated by the hot acidic phenol procedure (45). Eluted proteins were analyzed by SDS-polyacrylamide gel electrophoresis (8% polyacrylamide).

## RESULTS

### Bfr2 interacts with Dbp4 and Enp2

**Figure 2.**
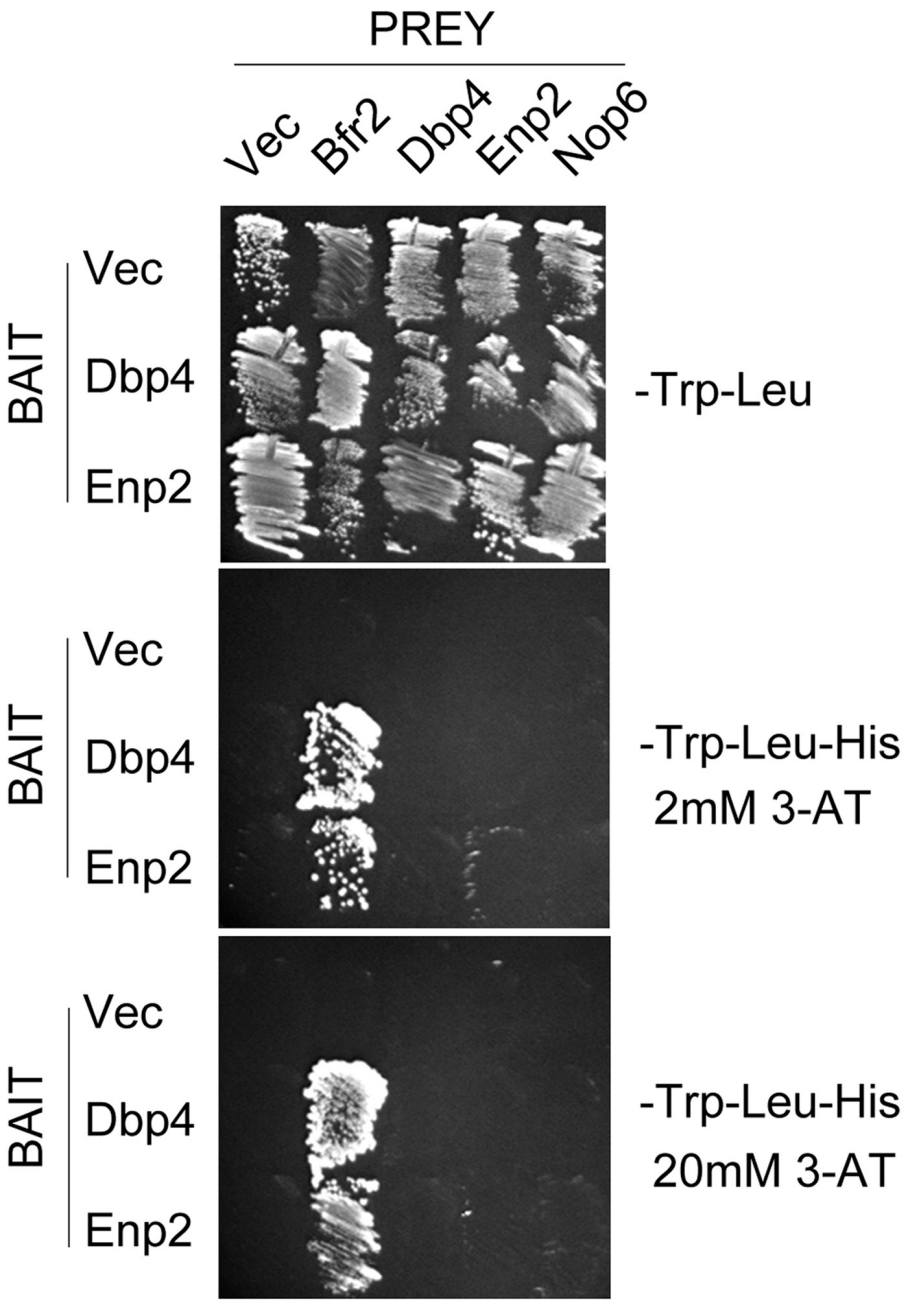
Directed yeast two-hybrid assays. Yeast strain AH109 was transformed with bait plasmid pGBKT7 (Vec) or its derivative pGBK-DBP4 or PGBK-ENP2, and prey plasmid pGADT7 (Vec) or its derivatives pGAD-DBP4, pGAD-ENP2, pGAD-BFR2 and pGAD-NOP6. The bait and prey plasmids, respectively, carry *TRP1* and *LEU2* auxotrophic markers that allow growth on medium lacking tryptophan and leucine (upper panel). Interactions between bait and prey hybrid proteins activate transcription of the *HIS3* reporter gene, which is monitored by growth on medium lacking histidine; addition of 2 or 20 mM 3-AT to this medium enhances the stringency of the *HIS3* reporter, allowing detection of the strongest two-hybrid interactions (middle and lower panels, respectively).

The function of many RNA helicases is likely modulated by interacting protein(s) (51). Our bioinformatics searches revealed that the C-terminal extension of Dbp4 harbors a coiled-coil motif that is conserved in all orthologs of Dbp4 (data not shown). This suggested that Dbp4 could interact with other protein(s) through its coiled-coil motif. To identify potential partners of Dbp4, we carried out extensive two-hybrid screens with yeast genomic libraries (46). Among the two-hybrid hits that were identified (unpublished data), Bfr2 was a very attractive candidate because it is a nucleolar protein that has a role in ribosome biogenesis (39). Database mining further suggested that Bfr2 was a likely partner of Dbp4, together with Enp2 (52–54). All three proteins are essential for yeast growth; they are phylogenetically conserved, and contain at least one coiled-coil motif (data not shown). Like Bfr2, Enp2 is a nucleolar protein that has been classified as a non-SSU processome component (39). We carried out directed two-hybrid assays using full-length Dbp4 as bait, and full-length Bfr2 and Enp2 as prey. We also included Dbp4 as prey because DEAD-box RNA helicases can function as dimers (30), and Dbp4 might do so as well (20). Controls with empty prey plasmid or empty bait plasmid (Figure 2) did not grow on selective media, ruling out a possible auto-activation of the reporter gene by the bait or preys. We used the ribosome biogenesis factor Nop6 as an additional negative prey control. We could not use Bfr2 as bait because it is an auto-activator (our unpublished observation). Cell growth was observed when Dbp4 (bait) was tested together with Bfr2, Enp2 or Dbp4 as preys on selective medium lacking 3-AT (data not shown). However, when adding 2 mM 3-AT to eliminate background activation of the *HIS3* reporter gene, or up to 20 mM 3-AT to increase the stringency of the selective medium (55), only the Dbp4–Bfr2 combination could grow (Figure 2), indicating that Dbp4 interacts more strongly with Bfr2 than with the other proteins.

Similar experiments were carried out using Enp2 as bait (Figure 2). Growth on selective medium was seen when Enp2 was co-transformed with either Bfr2 or Dbp4, but only the Enp2 and Bfr2 combination could grow in the presence of 20 mM 3-AT. This result indicates that Enp2 and Bfr2 strongly interact together (55). Our data are supported by previous studies that identified Bfr2 and Enp2 as potential partners of Dbp4, and Bfr2 as a potential partner of Enp2 (56–58). Taken together, our two-hybrid analyses suggest that the association between Dbp4 and Enp2 might be dependent on the presence of Bfr2.

### Dbp4 is associated with Bfr2 and Enp2 *in vivo*

**Figure 3.**
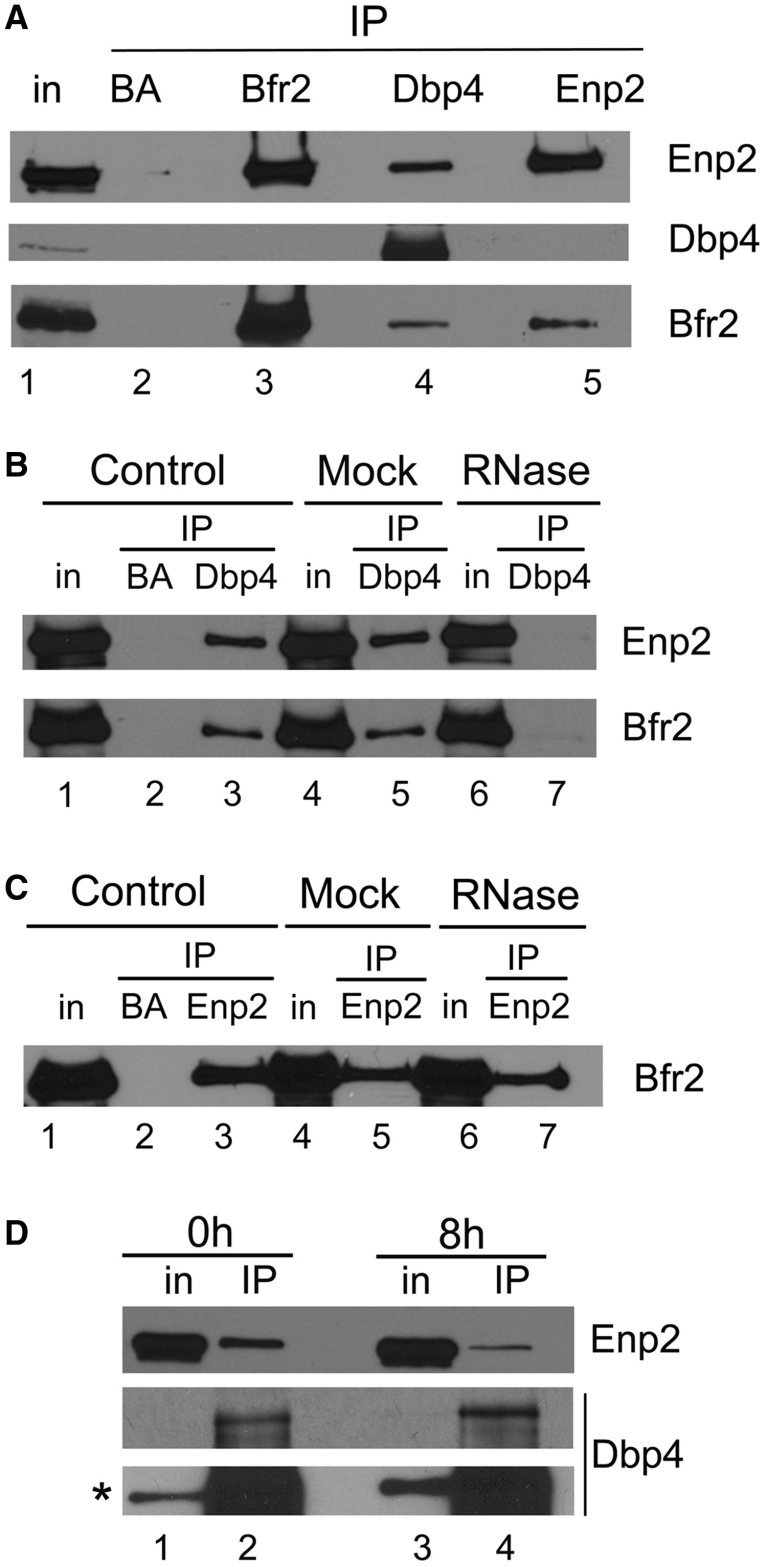
Analyzing interaction between Bfr2, Dbp4 and Enp2 by IPs. (**A**) Dbp4 associates with Bfr2 and Enp2 *in vivo*. IPs were carried out with anti-HA, anti-myc and anti-Dbp4 antibodies using extracts prepared from the double-tagged strain that expresses HA-tagged Bfr2 under the control of the *GAL1* promoter and myc-tagged Enp2 from its natural promoter. Control IPs were done in absence of antibodies (beads alone, BA). Lane 1 is whole cell extract (T is 6.5% input), and lanes 2–5 are IPs with beads alone (BA), anti-HA mAb (Bfr2), anti-Dbp4 antibodies and anti-myc mAb (Enp2). The same blot was subjected to immunodetection with various antibodies recognizing proteins identified on the right. (**B**) Dbp4 associates with Bfr2 and Enp2 in an RNA-dependent manner. Control IPs (lanes 1–3) were done as in Figure 3A. In the mock (lanes 4–5), the cellular extract was incubated at 37°C for 10 min before IP, and in lanes 6 and 7 the cellular extract was treated with RNase A for 37°C for 10 min. IPs were done in absence of antibodies (BA, lane 2) or with anti-Dbp4 antibodies (lanes 3–7), and immunoblotting was performed using anti-myc (Enp2) and anti-HA mAbs (Bfr2). T is 6.5% of input. (**C**) Association of Bfr2 with Enp2 is not RNA-dependent. IPs were carried out as in Figure 3B, except that anti-myc mAb (Enp2) was used for IP, and immunodetection was performed with anti-HA mAb (Bfr2). T is 6.5% of input. (**D**) Bfr2 is required for the association of Dbp4 with Enp2. Cellular extracts were prepared from undepleted cells (0 h, lanes 1–2) or Bfr2-depleted cells (8 h, lanes 3–4). IPs were carried out with anti-Dbp4 antibodies and western blotting analyses for Enp2 and Dbp4 were done with anti-myc mAb and anti-Dbp4 antibodies, respectively. The asterisk indicates the overexposed blot. T is 6.5% of input.

To validate the two-hybrid results, we verified the interaction between Dbp4, Bfr2 and Enp2 *in vivo*. We were not able to tag Bfr2 at its C-terminus (see also reference 39), therefore we generated a strain that expresses HA-tagged Bfr2 (HA-Bfr2) under the control of the *GAL1* promoter and myc-tagged Enp2 (Enp2-myc) from its natural promoter; this strain was named double-tagged strain. We carried out IPs with extracts prepared from the double-tagged strain grown in galactose-containing medium (Figure 3A). IPs were done using the anti-HA mAb for Bfr2 IPs, an anti-myc mAb for Enp2 IPs and rabbit polyclonal antibodies raised against Dbp4 (hereafter named anti-Dbp4). Control IPs were done with uncoated agarose beads (BA). These experiments show that Dbp4 is associated with Bfr2 and Enp2 *in vivo* (lane 4 in Figure 3A) and Bfr2 and Enp2 also interact together *in vivo* (lanes 3 and 5 in Figure 3A). Thus, IPs confirm the two-hybrid assay results, showing a strong interaction between Bfr2 and Enp2*.*

DEAD-box RNA helicases use the energy of ATP to bind and remodel RNA or RNA–protein complexes (34). We tested whether the association between Dbp4 and its two partners was dependent on the presence of RNA. IPs were carried out with cellular extracts pre-treated with RNase A. As shown in Figure 3B, the association of Dbp4 with either Bfr2 or Enp2 was lost when using RNase-treated extracts (compare lane 7 with lanes 3 and 5), showing that their association is RNA-dependent, in agreement with the recent demonstration that Dbp4 needs additional contacts with the extension flanking the RNA duplex for optimal helicase activity (60). In contrast, the interaction between Bfr2 and Enp2 was not affected by RNase treatment, showing that their association is not dependent on the presence of RNA (Figure 3C).

To determine if Bfr2 is required for the association of Dbp4 with Enp2, we carried out IPs with Bfr2-depleted cellular extracts. The double-tagged strain was grown to exponential phase in medium containing galactose (YPGal) and then shifted to dextrose-containing medium (YPD) for 8 h. We chose the 8-h time point for our experiments because western blot analysis showed no detectable Bfr2 in the cellular extract (Figure 5D, lower panel). Cells were collected from both culture media and IPs were done with anti-Dbp4 antibodies (Figure 3D). These experiments showed that the interaction between Dbp4 and Enp2 was decreased in Bfr2-depleted cells, and this was not due to loss of Dbp4 in the immunoprecipitate (Figure 3D, lower panel). These data corroborate our two-hybrid results, suggesting that Bfr2 bridges Dbp4 and Enp2.

### Bfr2 and Enp2 are necessary for early cleavages leading to 18S rRNA maturation

It has been shown that Dbp4 is necessary for early pre-rRNA cleavages at sites A0, A1 and A2 [(38); Figure 1]. Because Bfr2 and Enp2 associate with Dbp4, we decided to investigate their involvement in rRNA maturation.

**Figure 4.**
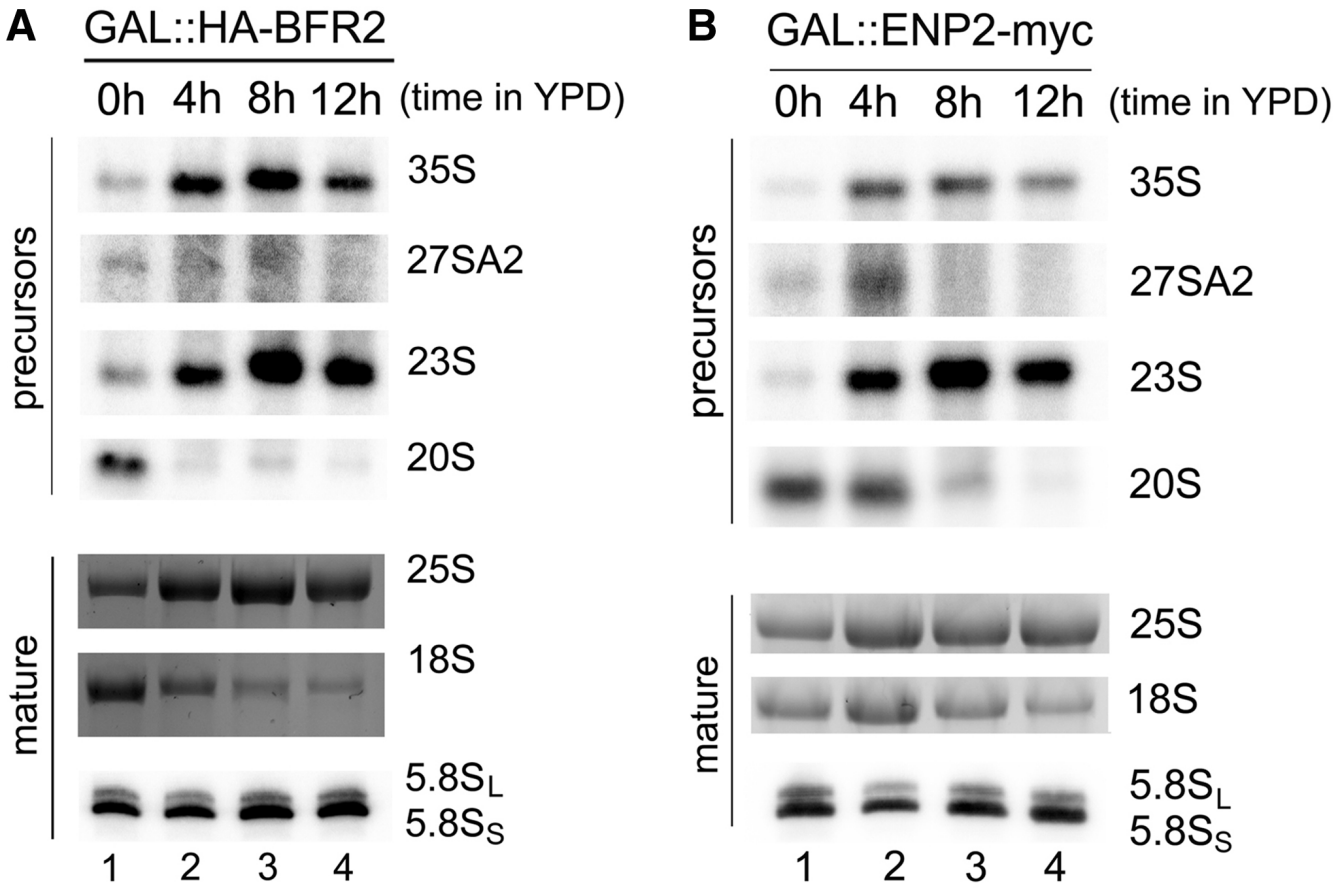
Bfr2 and Enp2 are required for pre-rRNA processing. Total RNA was extracted from depletion strains GAL::HA-BFR2 (**A**) and GAL::ENP2-myc (**B**) grown in YPGal (0h, lane 1), and at different depletion times after the shift in YPD (lanes 2–4). RNAs were analyzed by northern hybridization using probes directed against different rRNA precursors indicated on the right. Mature 18S and 25S rRNAs were visualized by staining with GelRed™. The short and long forms of 5.8S rRNA were detected by northern hybridization.

Cells were grown to exponential phase in YPGal using the following two strains: GAL::HA-BFR2 expressing HA-tagged Bfr2, and GAL::ENP2-myc encoding myc-tagged Enp2, both under the control of the *GAL1* promoter. The cells were then shifted to YPD and harvested at different time points after depletion; total RNA was extracted and used for northern analyses. Results of Bfr2 depletion are shown in Figure 4A: on depletion of Bfr2, there is a decrease in the production of the 27SA2 precursor, consistent with the loss of cleavage at site A2. We also observed an increase in the amount of 35S and 23S pre-rRNAs compared with the non-depleted sample. The 35S and 23S pre-rRNA usually accumulate in absence of early cleavages at sites A0–A2 (2). The levels of 20S pre-rRNA and the mature 18S rRNA were decreased, consistent with impaired cleavages at sites A0–A2. There were no changes observed in the abundance of the mature 25S and 5.8S rRNA. The same type of results were obtained with Enp2-depleted cells (Figure 4B): (i) high levels of 35S and 23S pre-rRNAs; (ii) low levels of 27SA2, 20S pre-rRNAs and mature 18S rRNA and (iii) no change in the levels of 25S and 5.8S rRNAs. Taken together these results indicate that Bfr2 and Enp2 are implicated in early processing events that lead to 18S rRNA production.

Polysome profiles of Bfr2- and Enp2-depleted cells were analyzed by sucrose density gradient sedimentation: we observed decreased amounts of 40S and 80S ribosomes, and an increase of free 60S subunits (data not shown). These defects are consistent with impaired 40S subunit biogenesis and the altered pre-rRNA processing events seen in Bfr2- and Enp2-depleted cells (Figure 4A and B).

### Bfr2 and Enp2 associate with the U3 snoRNA and Mpp10

**Figure 5.**
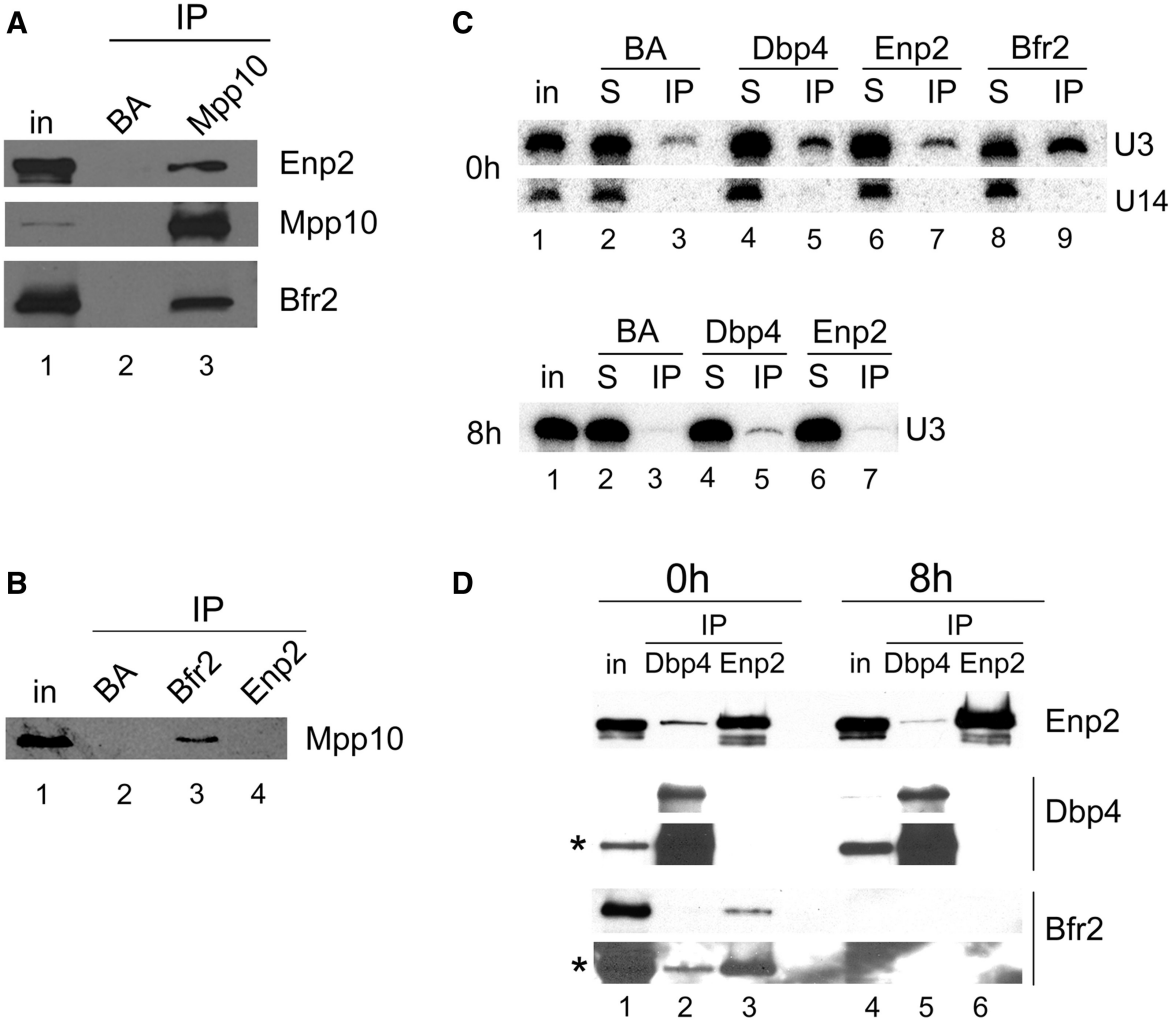
Bfr2 and Enp2 associate with Mpp10 and the U3 snoRNA. (**A**) Mpp10 associates with Bfr2 and Enp2. IPs were carried out with anti-Mpp10 antibodies, and immunoblotting was done with anti-myc (Enp2), anti-Mpp10 and anti-HA (Bfr2) antibodies. (**B**) Bfr2 interacts with Mpp10. IPs were carried out with anti-HA (Bfr2) and anti-myc (Enp2) antibodies and western blotting was done with anti-Mpp10 antibodies. (**C**) Association of U3 snoRNA with Bfr2, Dbp4 and Enp2 in presence or absence of Bfr2. IPs were carried out with beads alone (BA), anti-HA (Bfr2), anti-Dbp4 and anti-myc (Enp2) antibodies. Northern analysis was done with a radiolabeled oligonucleotide complementary to the U3 and U14 snoRNAs. In the top panel, cellular extracts were prepared from undepleted cells (0 h). In the bottom panel, cellular extracts were obtained form Bfr2-depleted cells (8 h). T is the input (10%), S is the supernatant (10%) and IP is the immunoprecipitated RNA. (**D**) IPs of Dbp4 and Enp2 in undepleted and Bfr2-depleted cells. IPs were done with undepleted (0 h) and Bfr2-depleted cells (8 h) using anti-Dbp4 and anti-myc (Enp2) antibodies, and immunoblotting was done with anti-myc (Enp2), anti-Dbp4 and anti-HA (Bfr2) antibodies. The asterisks indicate the overexposed blots.

We know that Dbp4 associates specifically with the U3 snoRNA and the U3-specific protein Mpp10 (our unpublished data), so we decided to verify if Bfr2 and Enp2 also associate with these SSU processome components. IPs were carried out with Mpp10 antibodies followed by western analysis (Figure 5A). The results show that Mpp10 associates with Bfr2 and Enp2. We also immunoprecipitated Bfr2 and Enp2, and observed that Bfr2 associates with Mpp10 (Figure 5B). The fact that Enp2 co-immunoprecipitates with Mpp10 but Mpp10 was not detected in Enp2 IPs suggests that the bulk of Enp2 is not in complex with Mpp10 or that the amount of co-immunoprecipitated Mpp10 is below detection limit. Nevertheless, these results show that Bfr2 and Enp2 can associate with Mpp10. To verify the association of the U3 and U14 snoRNAs with Bfr2, Dbp4 and Enp2, IPs were done using WCEs, as described in Figure 5B, followed by northern analysis (Figure 5C). The results indicate that Bfr2, Dbp4 and, to a lesser extent, Enp2 associate with the U3 snoRNA. There was no association between U14 snoRNA and Bfr2, Dbp4 or Enp2 (Figure 5C, upper panel). We then asked if the absence of Bfr2 affected the interactions between U3 snoRNA and Dbp4 or Enp2. Cellular extracts were prepared from the double-tagged strain after growth in YPD to deplete Bfr2, and IPs were done as described earlier. In the absence of Bfr2, the interaction between Enp2 and the U3 snoRNA was lost, whereas the association of Dbp4 with U3 was decreased about 2-fold (Figure 5C, lower panel). These data indicate that Bfr2 is necessary for the association of Enp2 with the U3 snoRNA. The absence of Bfr2 also affected the Dbp4–U3 snoRNA interaction (but to a lesser extent). Note that the efficiency of Dbp4 and Enp2 IPs with extracts from undepleted and Bfr2-depleted cells was the same (Figure 5D, upper and middle panel).

### Dbp4, Bfr2 and Enp2 associate with pre-rRNAs

**Figure 6.**
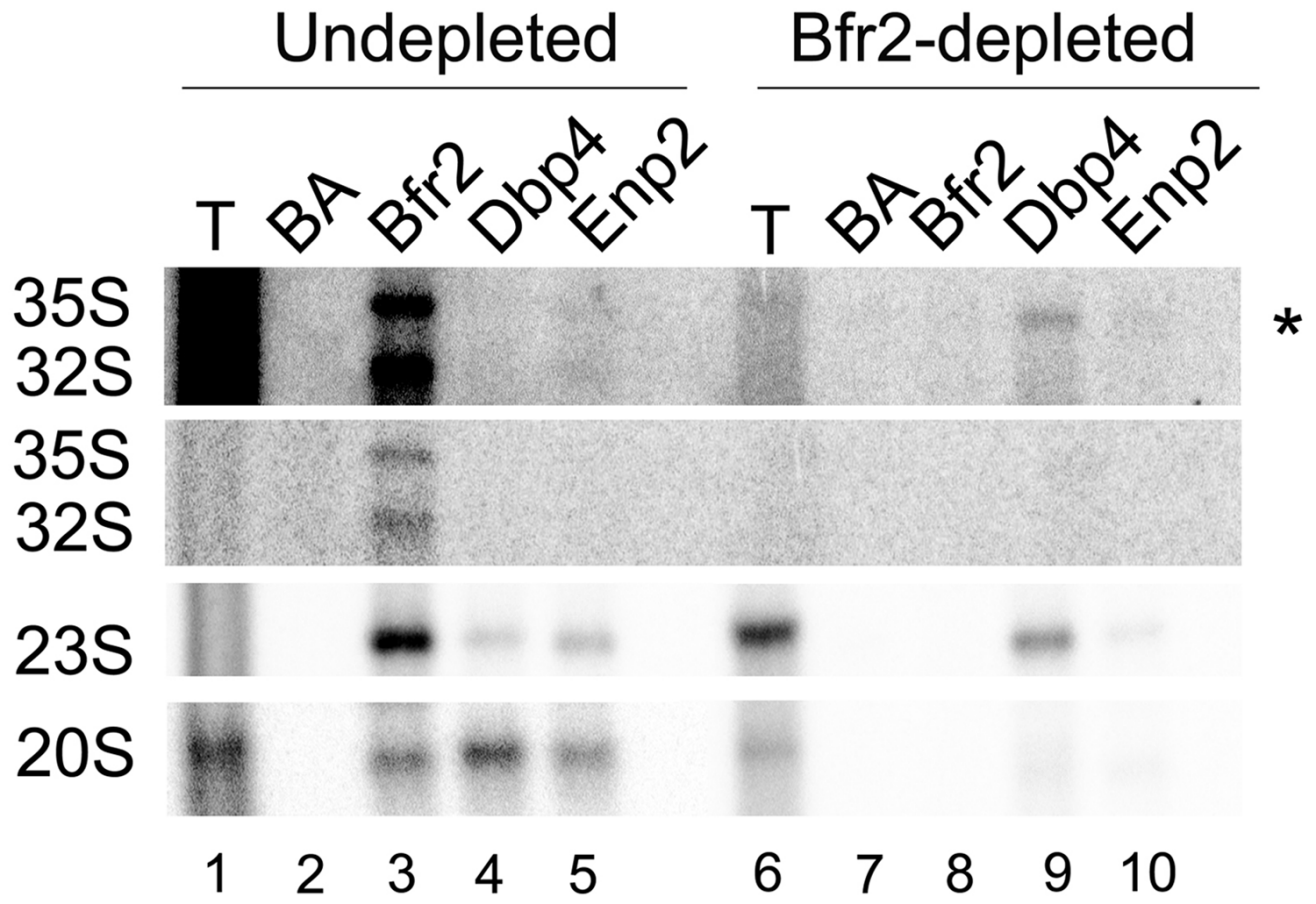
Bfr2, Dbp4 and Enp2 associate with pre-rRNAs. Cellular extracts were prepared from undepleted and Bfr2-depleted cells. IPs were done without antibodies (BA) and with anti-HA (Bfr2), anti-Dbp4 and anti-myc (Enp2) antibodies. Northern analysis was done with a radiolabeled oligonucleotide to detect pre-rRNAs. The asterisk indicates the overexposed blot.

Our results suggest that Bfr2, Dbp4 and Enp2 could be SSU processome components. To further investigate this possibility, we tested whether these proteins associate with rRNA precursors. Extracts were prepared from undepleted and Bfr2-depleted cells, and we carried out IPs followed by northern analyses (Figure 6). The results show that in the presence of Bfr2, the 23S pre-rRNA associates with Bfr2, Dbp4 and Enp2 (lanes 3–5). Interestingly, we observed that Bfr2, Dbp4 and Enp2 also interact with the 20S pre-rRNA (lanes 3–5). This result suggests that Bfr2, Dbp4 and Enp2 stay associated with the pre-rRNA after its cleavage at site A2. We were also able to detect the association of Bfr2 with the 35S and 32S pre-rRNA (lane 3). In the absence of Bfr2, there was a loss of association of Enp2 with the pre-rRNAs (lane 10). In contrast, Dbp4 remained associated with the 23S pre-rRNA, and to a lesser extent with the 35S pre-rRNA (see upper panel in Figure 6).

### Depletion of Bfr2 alters the sedimentation profile of Dbp4 and Enp2

**Figure 7.**
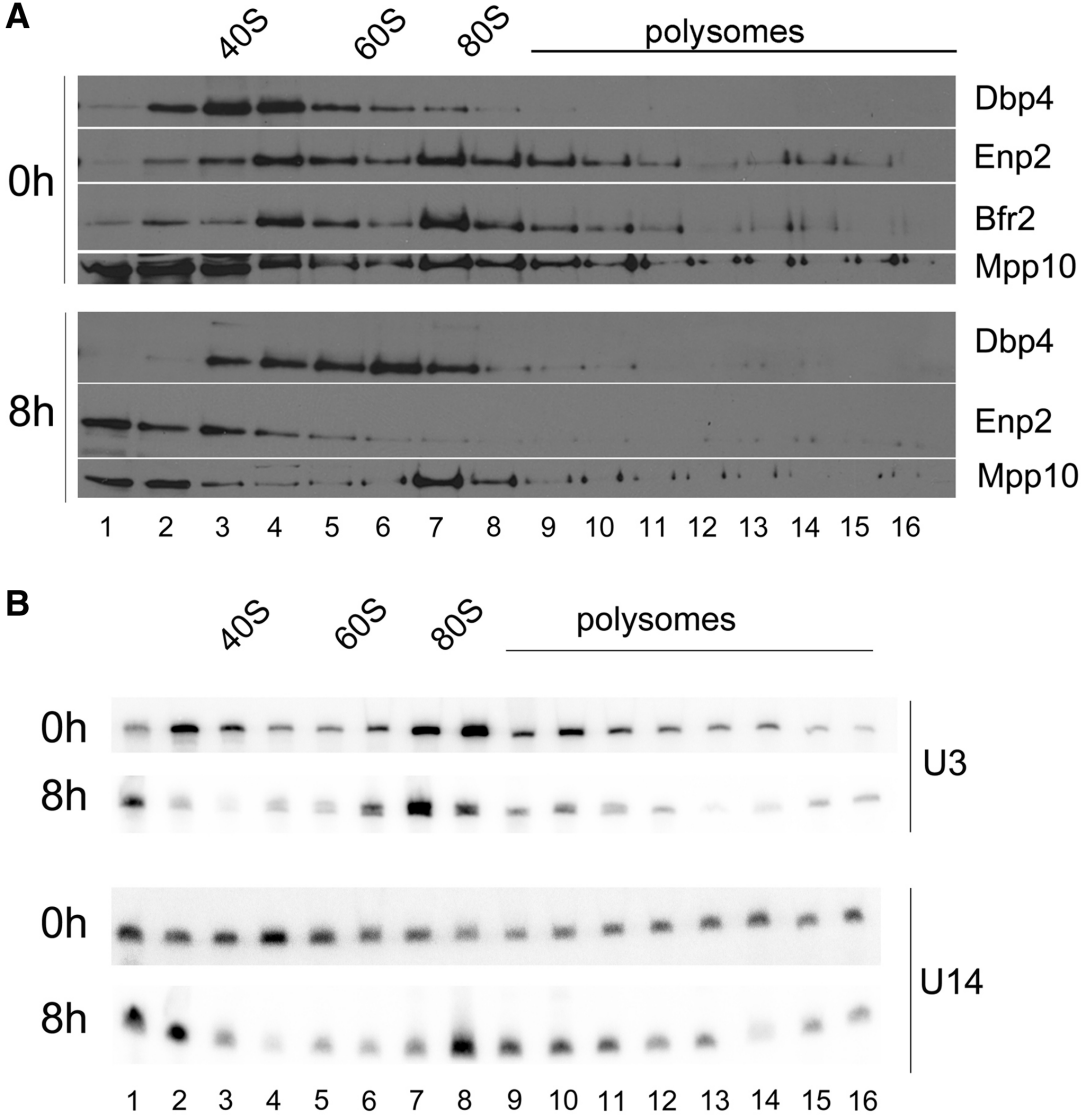
Sedimentation patterns on Bfr2-depletion. (**A**) Sedimentation profiles of Dbp4, Bfr2, Enp2 and Mpp10. Cellular extracts were prepared from undepleted (0h) and Bfr2-depleted cells (8h) and fractionated on 7–47% sucrose density gradients. Fractions 1–16 were subjected to western blot analysis using anti-myc (Enp2), anti-Dbp4, anti-HA (Bfr2) and anti-Mpp10 antibodies. The positions of 40S and 60S ribosomal subunits, 80S ribosome and polysomes are indicated. (**B**) Sedimentation profile of the U3 and U14 snoRNAs. Sucrose gradient fractions were prepared as in Figure 7A, except that RNAs were extracted from fractions 1–16 and subjected to northern blot analysis with radiolabeled oligonucleotides complementary to the U3 or U14 snoRNA.

We carried out sucrose gradient sedimentation analyses to determine the sedimentation behavior of Bfr2, Dbp4 and Enp2. The double-tagged strain was grown in YPGal and then shifted to YPD, and cellular extracts were prepared for ultracentrifugation through sucrose gradients. The gradients were fractionated into 16 fractions, and each fraction was subjected to western and northern analyses. As shown in Figure 7A, Dbp4, Bfr2 and Enp2 co-sediment in a peak of about 50S in sucrose gradients. Bfr2 and Enp2 are also enriched in the 80S region of the gradient, which contains very little Dbp4. The distribution of Dbp4 could reflect the transient nature of its interactions with component(s) of the 80S complex (see further text). We also analyzed the sedimentation profile of Mpp10, which was enriched at the top of the gradient and in the 80S region of the gradient. When cells were depleted of Bfr2 for 8 h, Dpb4 was distributed in a wide peak of 40–80S; the fact that Dbp4 appears in complexes of various sizes on depletion of Bfr2 implies that dynamic rearrangements of Dbp4 complexes require the presence of Bfr2. Depletion of Bfr2 also changed the sedimentation profile of Enp2, which sedimented in low-density fractions, suggesting that Bfr2 is required for association of Enp2 with complexes of about 50S and 80S. In contrast, the sedimentation profile of Mpp10 remained almost unchanged. These data indicate that depletion of Bfr2 alters the sedimentation profiles of Dbp4 and Enp2 but not that of Mpp10.

We also analyzed the sedimentation pattern of the U3 and U14 snoRNAs in the presence or the absence of Bfr2 (Figure 7B). The U3 snoRNA is normally detected in low-density fractions and in the 80S region of the gradient (top panel in Figure 7B). In the absence of Bfr2, there was no change in the overall sedimentation pattern of the U3 snoRNA (bottom panel in Figure 7B). This is similar to what was observed with Mpp10 in Bfr2-depleted cells (Figure 7A). However, there was an important change in the distribution pattern of U14 snoRNA with Bfr2-depleted extracts: U14 accumulated to a much higher extent in the 80S region, and this was accompanied by a decrease in its abundance in fractions 3–5 (Figure 7C). These results suggest that Bfr2 affects the release of U14 snoRNA from pre-rRNAs by Dbp4.

### Molecular interactions of Bfr2, Dbp4 and Enp2 in the 50S and 80S complexes

**Figure 8.**
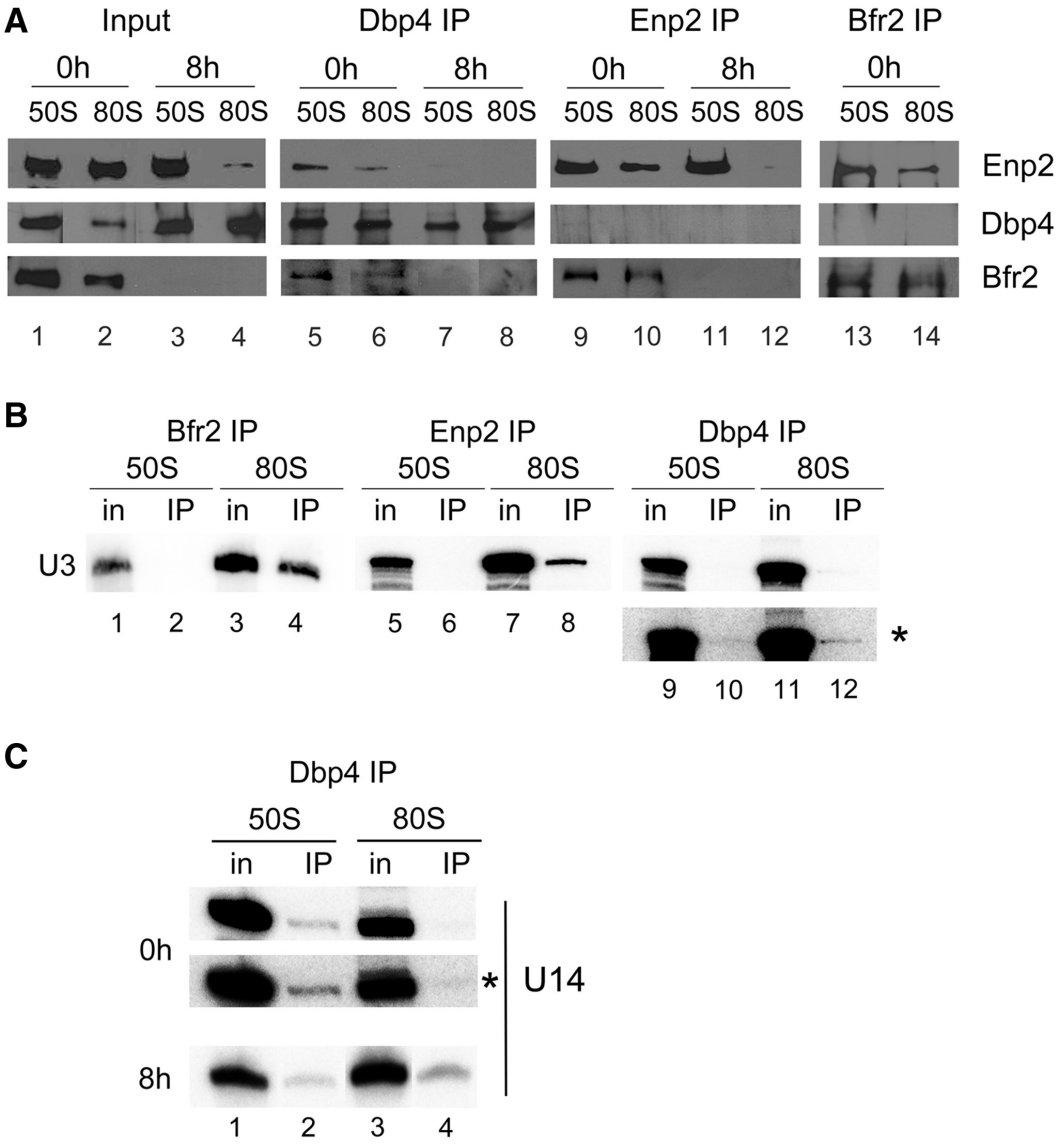
Association of Bfr2, Dbp4 and Enp2 in complexes of ‘50S’ and ‘80S’ isolated from sucrose gradients. (**A**) Cellular extracts obtained from undepleted and Bfr2-depleted cells were fractionated on sucrose gradients as in Figure 7A, and two series of inputs (In) were prepared for IPs: pooled fractions 3–5 correspond to the ‘50S’ complex and fractions 7–8 are the ‘80S’ complex. IPs were done with anti-Dbp4 (lanes 5–8), anti-myc (lanes 9–12), and anti-HA antibodies (lanes 13 and 14). Western blot analyses were carried out using the same antibodies to detect the presence of Enp2 (myc), Bfr2 (HA) and Dbp4. Input lanes correspond to 12% of pooled fractions. (**B**) Gradient fractions were prepared from undepleted cells and IPs were done as in Figure 8A, except that RNAs were extracted and subjected to northern hybridization with a radiolabeled oligonucleotide complementary to the U3 snoRNA. Inputs (In) correspond to 10%. The asterisk indicates the overexposed blot of Dbp4 IP. (**C**) IPs were done with anti-Dbp4 antibodies as in Figure 8B, except that northern hybridization was carried out with a radiolabeled oligonucleotide complementary to the U14 snoRNA.

We conducted a more refined analysis to investigate the association between Bfr2, Dbp4 and Enp2 in the 50S and 80S peaks. Sucrose gradient fractions were obtained from undepleted and Bfr2-depleted cells; fractions 3–5 (‘50S’ complex) or 7–8 (‘80S’ complex) were pooled together, and IPs were carried out on the 50S pool and the 80S pool followed by western blot analyses (Figure 8A).

The intensity of the signals in Bfr2, Dbp4 and Enp2 inputs from 50S and 80S peaks in undepleted and Bfr2-depleted cells correlated with their sedimentation profiles in sucrose gradients; for example, on Bfr2 depletion, the amount of Enp2 was reduced in the 80S peak compared with undepleted cells (compare lanes 2 and 4 in Figure 8A).

IPs with the ‘50S’ and ‘80S’ peak of undepleted cells revealed that Bfr2, Dbp4 and Enp2 co-precipitated (see lanes 5, 9, 13 and 6 in Figure 8A). These results suggest that Bfr2, Dbp4 and Enp2 associate together in the 50S and 80S peak. When Bfr2 was depleted, Dbp4 could no longer associate with Enp2 in the 50S and 80S (lanes 7and 8).

We investigated the association of the U3 snoRNA with Bfr2, Dbp4 and Enp2 in the ‘50S’ and ‘80S’ peaks. IPs were done as described in Figure 8A using undepleted cells and the U3 snoRNA was detected by northern hybridization (Figure 8B). U3 could be detected in the 50S peak but it did not co-immunoprecipitate with Bfr2, Enp2 or Dbp4. However, the U3 snoRNA present in the 80S peak (SSU processome) did co-immunoprecipitate with Bfr2 and Enp2, and to lesser extent with Dbp4 (detectable on overexposure; see the bottom panel with the asterisk). Thus, the ‘50S’ complex containing Bfr2, Dbp4 and Enp2 does not include the U3 snoRNA, but Bfr2, Dbp4 and Enp2 associate with U3 in the SSU processome.

We also verified if Bfr2, Dbp4 and Enp2 are associated with U14 snoRNA in the ‘50S’ and ‘80S’ peaks. There is no association between U14 snoRNA and Bfr2 or Enp2 in these peaks (data not shown). In contrast, U14 snoRNA was associated with Dbp4 in the ‘50S’ peak of undepleted cells, and in the ‘80S’ peak of Bfr2-depleted cells (Figure 8C). These results correlate well with the sucrose gradient sedimentation profiles (Figure 7C). In Bfr2-depleted cells, Dbp4 and U14 snoRNA remained associated in the 80S peak, suggesting the release of U14 snoRNA from the 80S complex was impaired in the absence of Bfr2.

**Figure 9.**
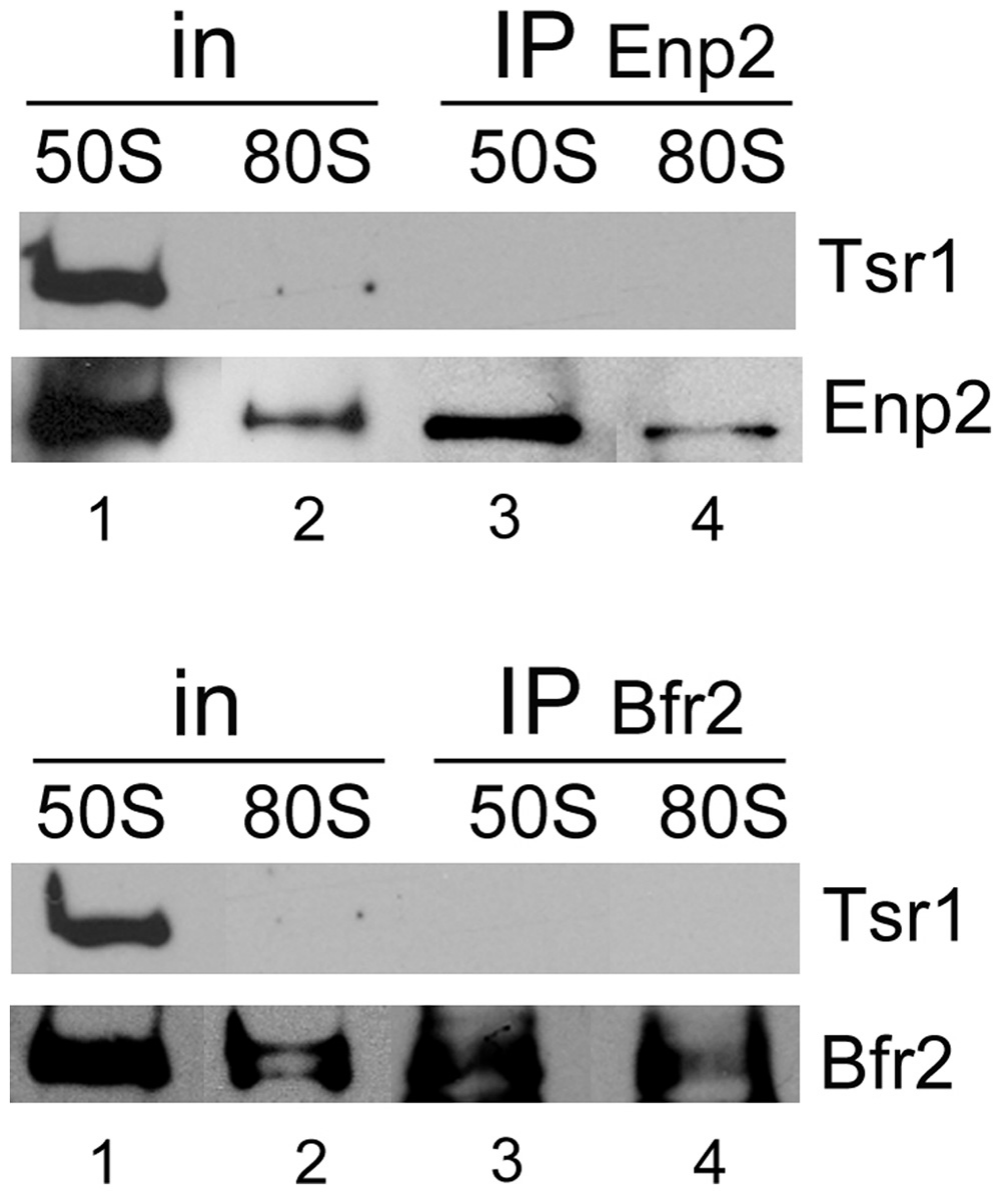
Bfr2 and Enp2 do not associate with Tsr1. IPs were carried out with anti-myc (Enp2; upper panel) and anti-HA (Bfr2; lower panel) mAbs as in Figure 8A, except that western blot analyses were done using anti-Tsr1 polyclonal antibodies.

To determine whether the ‘50S’ complex could be a pre-40S ribosome, we verified if Bfr2 and Enp2 were associated with Tsr1, a GTPase-like protein involved in assembly of pre-40S ribosomes (61,62). IPs conducted with the 50S and 80S peaks isolated from undepleted cells revealed that Tsr1 did not co-immunoprecipitate with Enp2, nor with Bfr2 (Figure 9). Therefore, the ‘50S’ complex containing Enp2 and Bfr2 is not a pre-40S ribosome.

### The binding partners of Bfr2

**Figure 10.**
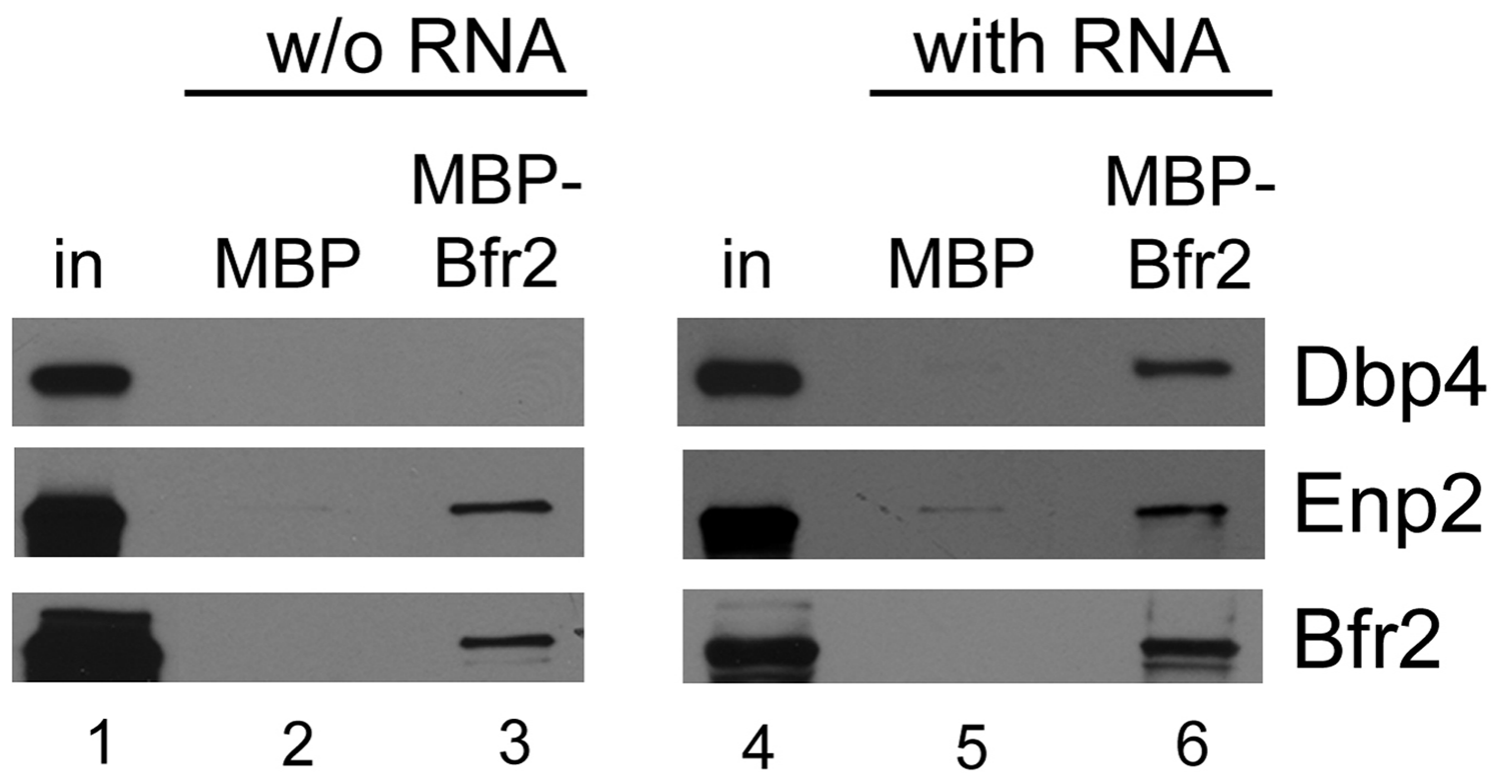
Pull-down assays with recombinant proteins. Pull-down experiments were carried out using MBP (lanes 2 and 5) or MBP–Bfr2 (lanes 3 and 6) bound to amylose beads. After incubation and elution, the presence of proteins Dbp4–His, GST–Enp2 and MBP–Bfr2 was detected by immunoblotting. Experiments were done in the absence of RNA (w/o RNA; left panels) or in the presence of yeast total RNA (with RNA; right panels).

To better define the nature of the interaction between Bfr2, Dbp4 and Enp2, we carried out pull-down experiments using bacterially expressed recombinant proteins. The results show that Bfr2 binds directly to Enp2 but not to Dbp4 (Figure 10, left panel). Adding Enp2 to the mixture did not improve Dbp4 binding to Bfr2 (data not shown). Interestingly, when yeast total RNA extracted with hot acidic phenol (and devoid of proteins) was added to the mixture, Dbp4 could bind Bfr2 (Figure 10, right panel). These results are in perfect agreement with our IP experiments, showing that association of Dbp4 with Bfr2 is RNA-dependent, and that the interaction of Enp2 with Bfr2 is not dependent on the presence of RNA (Figure 3).

### Association of U3 snoRNA with Mpp10 in depleted cells

**Figure 11.**
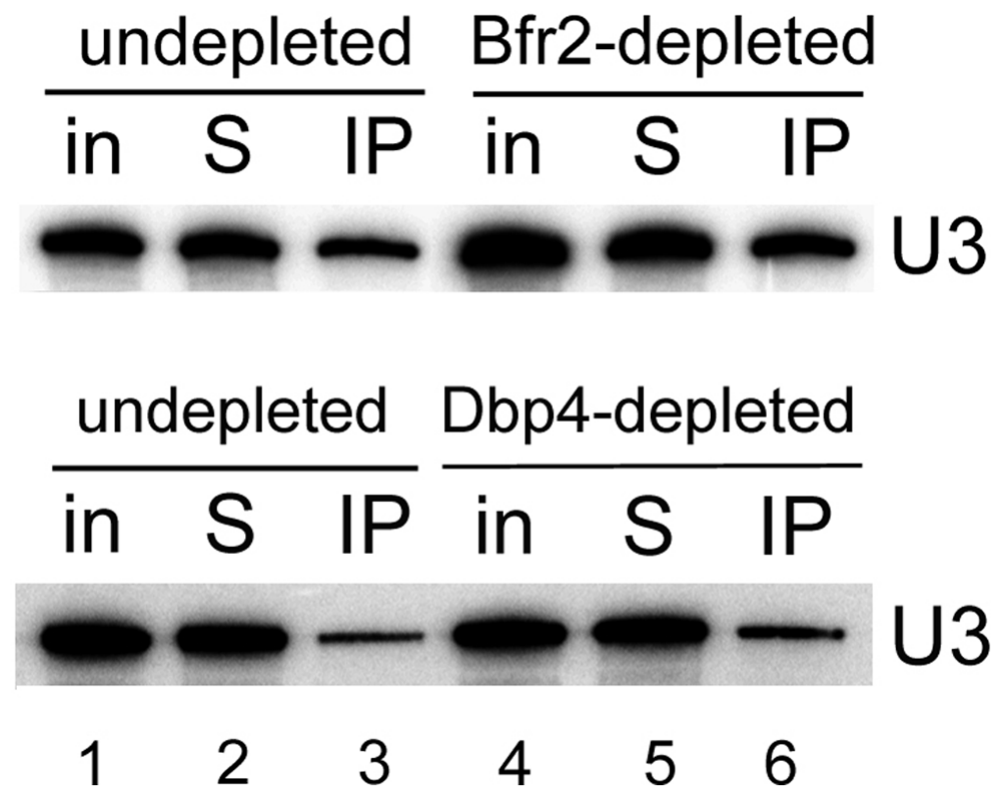
Association of U3 snoRNA and Mpp10 is not affected by depletion of Bfr2 or Dbp4. IPs with anti-Mpp10 antibodies were done with extracts from undepleted cells, or cells depleted of Bfr2 or Dbp4 for 8 h (as in Figure 3C).

To test the order of recruitment of Bfr2 and Dbp4 into the SSU processome complex, we determined whether the Mpp10–U3 snoRNA association was perturbed in the absence of Bfr2 or Dbp4 (Figure 11). These experiments showed that U3 snoRNA and Mpp10 remained associated in Bfr2- or Dbp4-depleted cells. Thus, our results suggest that Bfr2 and Dbp4 are recruited into the SSU processome after the incorporation of the U3 snoRNP and Mpp10 sub-complex.

## DISCUSSION

There are more than 200 non-ribosomal factors required for processing, modification and assembly reactions during ribosome biogenesis (1,6,9,26). A large number of these proteins are part of the SSU processome complex (14), which is necessary for the maturation of 18S rRNA (12,13). Some proteins of the SSU processome form specific sub-complexes (15–25), but more than a half of its components are not categorized into known sub-complexes (26). Moreover, most of the protein–protein interactions between SSU processome components have not been identified yet (26). Thus, studying the protein interactions of the SSU processome is important to refine our understanding of the assembly, architecture and activity of this complex during ribosome biogenesis (14,26). Dbp4 is one of the DEAD-box RNA helicases necessary for the early cleavages of the pre-rRNA at sites A0–A2, cleavages that lead to the production of 18S rRNA [(38); Figure 1]. To get a better understanding of the assembly and function of the SSU processome, we decided to analyze the role of Dbp4 in molecular interactions leading to the production of 18S rRNA.

We identified Bfr2 and Enp2 as partners of Dbp4 using yeast two-hybrid assays (Figure 2), and we showed by immunoprecipitation with antibodies to Dbp4 that Bfr2 and Enp2 associate with Dbp4 *in vivo* (Figure 3)*.* With the yeast two-hybrid system, there is always a risk that the bait protein binds a secondary factor that mediates (or bridges) the interaction with the prey protein. Pull-down assays with bacterially expressed recombinant proteins revealed that Bfr2 binds directly to Enp2 but not to Dbp4; however, when adding yeast total RNA to the mixture, Dbp4 could bind Bfr2 (Figure 10). The RNA used in these experiments is devoid of proteins, ruling out the possible involvement of a third protein mediating the interaction. As Bfr2 does not contain an RNA-binding motif, it is unlikely that RNA acts as a mediator of the interaction with Dbp4. Thus, the simplest explanation is that RNA binding to Dbp4 could induce a conformational change that facilitates its interaction with Bfr2.

When IPs were done *via* the Brf2 or Enp2 component, the results showed that Bfr2 and Enp2 interacted with each other, but not with Dbp4 (Figure 3A). It is possible that the amount of co-precipitated Dbp4 in IPs for either Bfr2 or Enp2 was under the detection limit. This may also reflect differences in the stoichiometry or differential accessibility of the tags within the complex. Depletion of Bfr2 impaired the association of Dbp4 with Enp2 (Figure 3D). Note that the association between Dbp4 and Enp2 was not completely lost, possibly because small amounts of Bfr2 could still be present after 8 h of depletion. Based on the results from two-hybrid assays, IPs and pull-down assays, we propose a model for the interaction between these three proteins. Bfr2 and Enp2 interact directly together in an RNA-independent manner (Figures 3 and 10). RNA binding to Dbp4 induces a conformational change, which allows interaction with Bfr2. In this scenario, Bfr2 would acts as a bridge between Dbp4 and Enp2.

Previous studies showed that Dbp4 is involved in the maturation of 18S rRNA. Our findings indicate that Bfr2 and Enp2 are also implicated in this process (Figure 4). In fact, the processing defects observed in either Bfr2- or Enp2-depleted cells are consistent with the involvement of Bfr2 and Enp2 in the early processing events at cleavage sites A0, A1 and A2. The hallmark of such processing defects is the strong accumulation of 23S pre-rRNA, which was observed in Bfr2- and Enp2-depleted cells (Figure 4). Li *et al.* (41) reported that Bfr2 and Enp2 are involved in pre-rRNA processing because their depletion led to accumulation of the 35S pre-rRNA; however, they did not see strong accumulation of 23S pre-rRNA on depletion. The phenotypes observed by Li *et al.* (41) could be due to degradation of 23S pre-rRNA on long depletion times [see also (39)].

Formation of the SSU processome is necessary for the maturation of the 18S rRNA (13). The SSU processome complex consists of the U3 snoRNA, Mpp10 (U3-specific protein) and many other nucleolar factors (12,14,39). Previous investigations indicated that Dbp4 associates with U3 snoRNA and Mpp10 (unpublished data), and here we showed that Bfr2 and Enp2 also associate with U3 and Mpp10 (Figure 5). We were able to co-immunoprecipitate Mpp10 with Bfr2 but not with Enp2 (Figure 5B) (although the amount of Mpp10 co-precipitated with Enp2 may be too small to be detectable by our western analyses). These analyses suggest that Dbp4, Bfr2 and Enp2 could be SSU processome components.

We showed that Bfr2, Dbp4 and Enp2 associate with various pre-rRNAs in non-depleted cells (Figure 6). Interestingly, these three proteins associate with the 20S pre-rRNA, suggesting that they remain associated with the rRNA precursor after the A2 cleavage until nuclear export, in line with the findings of Li *et al.* (41), who reported that Bfr2 and Enp2 were required for small subunit export. In Bfr2-depleted cells, the interaction of Enp2 with 23S and 20S pre-rRNA was lost. Thus, the presence of Bfr2 is required for the association of Enp2 with these pre-RNAs. In contrast, Dbp4 interacts with the 35S pre-rRNA and stays associated with the 23S pre-rRNA in Bfr2-depleted cells. Therefore, it appears that Bfr2 affects the molecular interactions of Dbp4 with pre-18S rRNAs during processing events leading to the maturation of 18S rRNA.

Analyzing the sedimentation pattern of ribosome biogenesis factors by sucrose gradient sedimentation is useful because co-sedimentation of non-ribosomal factors with the pre-ribosomal particles may suggest physical interaction with these particles. The SSU processome has a sedimentation coefficient of about 80S (12). The data obtained from sucrose gradient sedimentation and IPs on pooled fractions of the gradient (Figure 7 and 8) indicate that the 50S complex contains Bfr2, Dbp4, Enp2 and U14 snoRNA (and possibly additional nucleolar factors), whereas the 80S complex (the SSU processome) contains Bfr2, Dbp4, Enp2 and U3 snoRNA (Figure 8A and B). These results highlight the dynamic reorganization of large complexes during maturation of 18S rRNA. Note that the ‘50S’ complex is not a pre-40S ribosomal particle, as there was no association of Tsr1 with Bfr2 or Enp2 (Figure 9). It has been shown that actinomycin D treatment induces accumulation of a 50S U3 snoRNP particle that contains DDX10 (human homolog of Dbp4) in HeLa cells (63). Given that the ‘50S’ complex described in this study does not contain the U3 snoRNA, it appears that the 50S complex seen in HeLa cells is not the same as the one we characterized here.

Another interesting observation was that when using WCEs for IPs, there was no association between U14 snoRNA and Bfr2, Dbp4 or Enp2 (Figure 5C). However, Dbp4 was associated with U14 snoRNA in the ‘50S’ peak in undepleted cells (Figure 8C). When cells were depleted of Bfr2, U14 snoRNA was associated with Dbp4 in the 80S peak, suggesting that Bfr2 could be implicated in the release of U14 snoRNA from the ‘80S’ complex. Discrepancies between IPs with WCEs and sucrose gradient fractions could be explained by the amount of material used, or simply by the fact that fractions isolated from sucrose gradients are partially purified complexes, which may enhance the efficiency of IPs by eliminating interfering components.

Based on the following data, we propose that Bfr2, Dbp4 and Enp2 are SSU processome components: (i) Bfr2, Dbp4 and Enp2 are nucleolar proteins; (ii) they are involved in pre-rRNA processing at cleavage sites A0, A1 and A2; (iii) these proteins associate with the U3 snoRNA and Mpp10, and also interact with different pre-18S rRNA species and (iv) Bfr2, Dbp4 and Enp2 co-sediment in a peak of about 80S, and they associate with U3 snoRNA in that peak.

**Figure 12.**
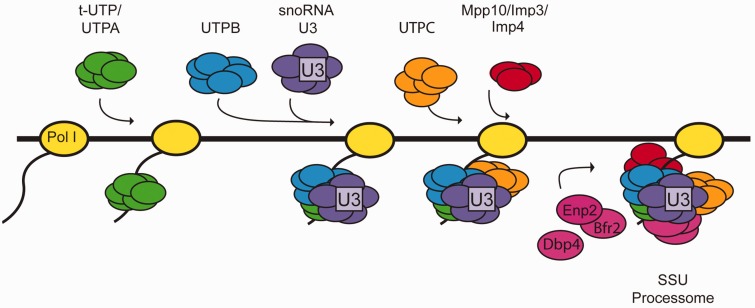
Simplified model for the assembly steps of the SSU processome. (i) The UtpA/t-Utp sub-complex assembles on the 5′ETS of the nascent pre-rRNA; (ii) UtpB then associates with the pre-rRNA followed by binding of the U3 snoRNP complex (iii); (iv) UtpC interacts with the pre-rRNA independent of UtpB and U3 snoRNP; (v) Mpp10 sub-complex assembly takes place after U3 snoRNP binding; (vi) The Bfr2–Enp2 dimer and Dbp4 incorporate the SSU processome particle.

Nan1 is a component of the t-UTP sub-complex, which assembles at a very early step during SSU processome formation (even before U3 snoRNP incorporation). Depletion of Nan1 changes the sedimentation profile of U3 snoRNA (27). The absence of Bfr2 did not alter the sedimentation profile of the U3 snoRNA and Mpp10 (Figure 7). Moreover, the absence of Bfr2 or Dbp4 did not affect the association between U3 snoRNA and Mpp10 (Figure 11). These results suggest that Bfr2 and Dbp4 might assemble into the SSU processome after assembly of the U3 snoRNP and Mpp10 sub-complexes. According to our results and the studies of (28,64), we propose a simplified model for the assembly steps of the SSU processome (Figure 12). First the UtpA/t-Utp binds to the pre-rRNA followed by two mutually independent steps: one step includes the assembly of the UtpB and the other involves the association of UtpC with the 35S during later assembly steps. The U3 snoRNP base pairs with the pre-rRNA after UtpB binding, which in turn allows the assembly of the Mpp10 sub-complex on the nascent pre-rRNA. Bfr2, Enp2 and Dbp4 then incorporate into the SSU processome particle. The results from IPs and sucrose gradient sedimentation, combined with the observation that the absence of Bfr2 did not affect the association of Dbp4 and U3 snoRNA in the 80S peak (data not shown) suggest that Bfr2 and Enp2 are recruited together but Dbp4 could be incorporated independently from Bfr2 and Enp2.

Our results provide new insight into the order of assembly of three nucleolar proteins into the nascent SSU processome. These additional data refine our understanding of SSU processome structure and function.

## FUNDING

The National Sciences and Engineering Research Council of Canada (NSERC), [RGPIN 249792]. Funding for open access charge: NSERC

*Conflict of interest statement*. None declared.
